# **Author index to Volume 83**

**DOI:** 10.1054/bjoc.2000.1591

**Published:** 2000-12


					Aaltonen L 1643
Aaltonen LA 1015
Aapro MS 1637
Aasheim H-C 743
Abbou CC 209
Abdulkarim B 514
Abe T 887
Abecassis J 1380
Abels C 1216
Abra RM 232, 684
Actane Consortium 1654
Adami H-O 949
Adams J 561
Agudo A 104
Ahern R 980
Aherne GW 146, 792
Ahlbom A 692
Ahmed A 127
Ahmed T 360
Aikou T 701
Akagi K 887
Akahira J 1488
Akazawa K 986
Akedo H 769
Akhmedkhanov AA 1255 (L)
Akinaga S 1039
Akisik E 737
Alberto P 1637
Albo D 298
Albright RE 588
Alemi M 307
Alexander FE 487
Alexopoulos CG 1128
Alho O-P 614
Ali SA 1061
Allal AS 1637
Allen DG 1659
Allen MJ 980
Allen NE 95
Allman R 655
Altomonte V 1405
Altorki N 493
Alvarez B 526
Amato R 1256 (L)
Amino N 1495
Amir Z 284
Andersen TI 1650
Anderson DQ 1061
Anderson E 992
Anderson EDC 98
Anderson H 447
Anderson JJ 498
Anderson TJ 487
Andersson S 307
Andoh A 769
Andrion A 104
Andrulis IL 737
Angeletti CA 480
Anglian Breast Cancer Study
Group 1301
Angus B 498
Anselmi L 1412
Anthoney A 426
Anthuber M 1216
Appleby PN 95
Aquino-Parsons C 1525
Araki K 609
Araki T 196
Arcangeli A 1722
Archimandritis S 1281
Arends J-W 252
Argiles JM 526
Aridome K 701
Armatis P 906
Armes JE 1659
Armstrong DK 1405
Ascierto PA 1707
Ashley S 91, 1418
Asselain B 1480
Atzpodien J 583
Auclerc M-F 1617
Aurlien E 1375
Austrup F 1664
Autier P 1243
Auvinen A 956
Awad L 431
Awwad HK 30
Ayude D 1139
Baba H 141, 1168
Baccino FM 526
Bachellerie-Rhein 1594
Bader S 1646
Bailey D 421
Baird MS 914
Balkwill FR 1538
Baniel J 463
Banks E 412
Barak M 1696
Barber MD 1443
Barberi-Heyob M 1380
Barbet N 594
Barbier P 544
Barbone F 1238
Barfoot R 177
Baron PL 16
Baronius W 458
Barraclough R 1084, 1473
Barret J-M 1740
Barth S 252
Baruchel A 1617
Baskaran R 1360
Basolo F 480
Bastuji-Garin S 209
Bate S 980
Bates SE 817
Baudin E 715
Baum M 1769 (BR)
Baumann P 1387
Baumohl J 1128
Bax CMR 1730
Bay BH 319
Becchetti A 1722
Begg AC 899
Beijnen J 375
Beketic-Oreskovic L 892
Belometti MO 573
Belzile E 112
Ben-Eliyahu S 1630, 1747
Benasso M 1437
Benedict WF 1039
Benepal TS 146
Bengisu E 737
Beretta GD 573
Bergenheim AT 826
Bergerat J-P 346
Bergeron C 973
Berghmans T 1128
Bergman L 1003, 1009
Bergstrom R 391
Berner A 743
Berney CR 1637
Berrington A 412
Berry C 1047
Besret C 1309
Beuzeboc P 1480
Bicknell R 63
Bicocchi MP 1295
Bilbao J 847
Binns W 1565
Birch JM 467, 1136
Birchall MA 421
Bischoff P 642
Bishop MC 443
Bissery MC 544
Bitisik O 737
Bjellerup P 171
Black RJ 387
Blagosklonny MV 817
Blake C 1330
Blakey DC 267
Bleiberg H 431
Blok LJ 246
Bockmann B 1664
Boehm J 473
Boivin J-F 112
Bokemeyer C 458
Boldrini L 480
Bolger B 566
Bombardelli E 1762
Bonaiti-Pellie C 177
Bond MG 447
Bond S 1405
Boothroyd C 1003
Bordigoni P 1617
Borg A 775
Borrani E 1722
Borresen-Dale AL 1650
Borri P 1722
Borst P 366, 375
Bosetti C 689
Bosserhoff A-K 1216
Botti G 1707
Botting B 278 (L)
Bouffler SD 1291
Bourhis J 514
Boutard P 973
Boven E 921
Bower M 1432
Bowman A 98
Bown NP 40
Bowskill C 935
Boyano MD 847
Boyer M 438
Boyle JM 467, 1136
Brada M 588
Bradshaw TD 270
Brannan M 1599
Brattsand G 311
Braybrooke JP 219
Breitkreutz D 1387
Brekelmans CTM 384
Brewster DH 387
Bright E 1405
Brinkmann AO 246
Britton JA 1552
Brodie AM 74
Bromelow KV 853
Broome P 443
Brossault S 1418
Brown JE 1448
Brown KW 1583
Brown NJ 1061
Bruchim Bar Sade R 153
Bruland OS 1375
Bruner J 588
Buccoliero AM 1722
Buer J 583
Buettner R 1216
Bugat R 431
Burchell J 1202
Burt EC 467
Burt PA 447
Busam KJ 493
Author index to Volume 83
Key to abbreviations:
(BR) Book reviews; (L) Letters to the editors
1771
British Journal of Cancer (2000) 83(12), 1771-1777
(c) 2000 Cancer Research Campaign
doi: 10.1054/ bjoc.2000.1591, available online at http://www.idealibrary.com on
Busch R 35
Bush JA 1468
Busquets S 526
Bussink J 674
Butow PN 1448
Bykovskaia SN 506
Cabioglu N 737
Cagini L 569
Cai Q 506
Cailleux AF 715
Calcinai A 480
Caldas C 1309
Calderwood MA 329
Calleja A 104
Calogero A 184
Calsou P 514
Calvete JA 267
Cameron D 1009
Cameron DA 98
Cameron DW 134 (L)
Campbell MJ 959
Canavate ML 847
Capdeville R 594
Cappello M 569
Carbo N 526
Cardinal J 1009
Cardinali B 1503
Carestia L 8
Carmichael J 447
Carothers A 1643
Carozzi F 1462
Carpi A 1412
Carthew P 935
Castello G 1707
Castiglione F 707
Casula M 1707
Cazenave J-P 346
Cerignoli F 1503
Cesarini J-P 1243
Chadha-Ajwani S 246
Chamoun E 1243
Chang A 750
Chaplin DJ 811
Chapman AD 632
Chard T 1730
Chastagner P 973
Chatzaki E 1730
Chatzistamou I 906
Cheeseman SL 1599
Chellini E 104
Chen C-L 1096, 1674
Chen GK 892
Chen J-Y 1674
Chen Y-T 493
Chen Z 1367
Cheng A-L 1510
Cheng KK 127
Cherian SP 83
Cherubini A 1722
Cheung Jr K-JJ 1468
Chia S-J 761
Chiao JW 360
Chilvers CED 127
Chin K-F 1425
Chin S-F 1309
Chisamore MJ 782
Choi L 594
Chompret A 177
Chopin DK 209
Chott A 454
Chow VTK 319
Christen RD 1360
Christmas TJ 1274, 1432
Christofori G 1
Chua M-S 270
Ciatto S 1462
Cigolari S 707
Ciolino HP 333
Clark IM 1147
Clarke B 1405
Clarke NW 992
Clarke RB 992
Clemens M 458
Coates AS 1448
Coche-Dequeant B 1594
Cocker HA 338
Coelho D 642
Cole DJ 16
Collet J-P 112
Collins N 83
Colombino M 1707
Colpaert F 1516
Colston KW 239
Compagni A 1
Confortini M 1462
Connolly CK 447
Conte M 1412
Cook-Mozaffari P 127
Cooke T 566
Cooper GS 404
Cooper RA 620
Coplan K 493
Coralli C 662
Cordero OJ 1139
Cornelisse CJ 719
Cornu G 1617
Cortes RE 969 (L)
Corvo R 1437
Cossu A 1707
Costelli P 526
Cotterill SJ 397
Cottier B 447
Couillault G 973
Courtay-Cahen C 1309
Courtney M 1454
Cowburn P 655
Cox R 1291
Coze C 973
CPT-11 F205, F220, F221 and
V222 study groups 431
Craft AW 397
Crociani O 1722
Croke DT 725
Crowl-Bancroft CV 1367
Csanady M 261
Cui X 50
Culine S 431
Culjak G 1448
Cullen MH 1623
Cunningham D 146
Cunningham DC 287
Curtin NJ 267
Cuzick J 561
Dachs GU 662
Dagan E 153
Dalay N 737
Daley FM 30
Dalgleish AG 239
Dalmasso P 104
D'Amico M 1437
Danesh J 970 (L)
Darbre PD 1183
Darby S 412
Daures J-P 8
Davey GK 95
David K 1747
David S 544
Davidson JM 1309
Davidson NE 1405
Davidson SE 620, 1702
Davies P 561
Davies S 1730
Davis JM 1405
Day N 121, 692
Day NE 127
de Bock GH 719
de Bono JS 426
de Haas M 366, 375
De Raucourt D 1594
de Rooij FWM 539
De Stavola BL 964
de Tourreil S 177
de Vries EGE 594
de Wilde PCM 674
de Wilt JHW 1176
Deacon JM 1565
Dearnaley DP 1623
Defachelle AS 973
Del Governatore M 1544
del Mar Melero-Montes M 1556
Deming SL 1688
Denhardt DT 156
Deo H 412
Deplanque G 346
Derby LE 1556
Desal M 1565
Deutsch E 514
Devalck C 973
Devilee P 719
Dhanabal M 1077
Di Marcotullio L 1503
Dias S 1538
Diaz-Perez JL 847
Diaz-Ramon L 847
Dickens D 1599
Dickson RB 1688
Dictor M 775
Didelot C 1380
Dieras V 1480
Dincer M 737
Dinsdale D 935
Distefano M 1762
Dockerty J 692
Doherty AP 1432
Domenge C 1594
Donadieu J 1617
Dong C 407
Dong G 1367
Donnelly CA 134 (L)
Doong S-L 1674
Dore J-F 1243
Dorval T 1454, 1480
dos Santos Silva I 964
Doweck I 1696
Dragosics B 454
Driesel G 1664
Droz J-P 431
Duffey DC 1367
Duffy S 121
Dufour P 642
Dumez H 594
Dunican D 725
Dunlop MG 1643
Dunn SM 1448
Duran GE 892
Durand RE 1525
Durmuller U 204
Duthie SJ 1532
East SJ 267
Eder C 1664
Edixhoven A 539
Edwards DR 1147
Edwards PAW 1309
Eggermont AMM 6, 1176, 1351
Einatan J 761
Eisen T 980
Ekbom A 949
Ekstrom AM 391
Ekstrom TJ 1020
Ellershaw C 1124
Elsasser-Beile U 637
Engelstein D 463
Engert A 252
English MA 550
EORTC Melanoma Co-operative
Group 1243
Erikstein B 1650
Esche C 506
Eschwege F 514
Escolar A 104
Esmailzadeh A 1249
Espie M 577
Etievant C 1516
Eu K-W 750
European Organization for
Research and Treatment of
Cancer 1351
Evans CD 1565
Evers R 366, 375
Extra J-M 1480
Fahimi S 1249
Falk SJ 447
Farahvash MJ 1249
Farmery SM 188
Farrington SM 1643
Farris A 707
Faulkner SW 729
Fearon KCH 1443
Felletti R 707
Fellous A 544
Fenwick F 498
Ferguson JSJ 1268
Ferlini C 1762
Ferrari P 1412
Ferrier CM 1351
Fetting J 1405
Feychting M 692
Fezoulidis I 1281
Field WN 56
Figer A 153
Fink K 588
Fink U 473
Floderus B 1231
Florin MC 1128
Floris C 1707
Flux GD 287
1772 Author index
British Journal of Cancer (2000) 83(12), 1771-1777 (c) 2000 Cancer Research Campaign
Fodde R 1291
Fontana L 1722
Fontanini G 480
Foote D 278 (L)
Ford HER 146
Formanek M 454
Forouhi P 98
Forrest APM 487
Fortier I 1559
Fossa A 743
Fossa SD 743, 1623
Foulkes W 177
Fourquet A 1318
FRALLE Group 1617
Franceschi S 689, 1238
Franzke A 583
Fraser LA 387
Frater A 561
Frati L 1503
Fredericks R 588
French Acute Lymphoblastic
Leukaemia (FRALLE) Group
1617
French Group d'Etude des
Tumeurs de la Tete et du Cou
(GETTEC) 1594
Freneaux P 1318
Frey M 1338
Freyer G 431
Fridman WH 1454
Friedlander ML 729
Friedman E 153, 463
Friedman H 588
Frisch M 89
Fritsche H 1256 (L)
Frontini L 707
Fujii H 1607
Fukuda R 1394
Fukuda T 1168
Futamura N 1681
Gallagher CJ 1612
Gallo C 707
Gammon MD 1552
Ganesan TS 219
Gao F 699 (L)
Garcia de Galdeano A 847
Garcia-Vazquez MD 847
Gardeazabal J 847
Gardiner E 1425
Gardner C 1599
Garland SM 1659
Gasparini G 707
Gaudernack G 743
Geloen A 1055
Gem Vin Investigators 707
George NJR 992
George SL 959
Gershoni-Baruch R 153
Gescher A 935
GETTEC 1594
Ghazizadeh M 196
Giaccone G 1069
Giachi M 1722
Giannini G 1503
Giesing M 1664
Gil-Diez de Medina S 209
Giles MS 329
Giokas G 1234
Glantz M 588
Glendon G 737
Goepel JR 1061
Goh H-S 761
Goldstraw P 569
Gonzalez CA 104
Goodwin DJ 329
Goodwin S 519
Gordon RJ 22
Gore ME 980
Gorman T 1084
Gornall P 602
Gorringe KL 1309
Goto S 1096
Gounon P 544
Graham J 426
Grant G 1532
Gray SG 1020
Grdina W 782
Greaves MJ 467, 1136
Green J 412
Greenberg E 1696
Greenberg H 588
Greenman J 1425
Gregory K 853
Gregory WM 98
Gridelli C 707
Grigoryev DN 74
Grill H-J 1664
Grimmond S 1003
Gritti G 573
Grivegnee A 1243
Groen HJM 594
Grondin A 998
Grothey A 870
Groupe d'Etude des Tumeurs a
Calcitonine 715
Groves F 956
Gruia G 146
Grussenmeyer T 637
Guarneri D 1437
Guba M 1216
Gudat F 204
Guillou PJ 188
Gulino A 1503
Gulisano M 707
Gumbrell L 1599
Gurney H 438
Haag C 458
Hain SF 863
Hakulinen T 956
Hall KT 329
Hamblin MR 1544
Hamilton G 454
Han EK-H 83
Hanley JA 112
Hansson L-E 391
Harani N 941
Hardcastle A 792
Hardy R 964
Harland SJ 1623
Harper PG 863
Harrington KJ 232, 684
Harris AL 63, 219
Harrison D 1646
Hart SL 662
Hartley L 1003
Hartmann JT 458
Hartmann O 973
Harvey P 1147
Hasan T 1544
Hasegawa Y 1495
Hashizume R 870
Haslett C 941
Hatano K 324
Haugk B 498
Haustermans K 899
Haward RA 284
Hayashi H 1209
Hayes AJ 1154
Hayflick L 841
Hayward N 1003, 1009
Hazama S 1026, 1209
Heberer M 204
Helmerhorst TJM 246
Hemminki K 407
Hempel V 458
Hendry JH 1702
Henriksson R 826
Henry-Amar M 577
Herait P 431
Hernandez S 104
Heron J-F 577
Herrero R 1238
Herrmann R 1454
Hess AD 1405
Hewison M 550
Higashiyama T 1495
Hilinski J 1047
Hill BT 1516, 1740
Hill C 1594, 1617
Hill SA 811
Hinnen P 539
Hiraoka S 668
Hirose H 1681
Hirose K 1026, 1209
Hjelm NM 1330
Ho CS 1330
Ho SKW 1330
Hoelscher AH 473
Hoffmann R 583
Hofland I 899
Hofland N 1291
Hohl RJ 588
Hohmann B 756
Holder AL 811
Holdsworth H 1538
Hole D 566
Holl V 642
Holm NV 1231
Hoogenboom HR 252
Hoos A 1323
Hopster D 1287
Hopwood P 447
Horikoshi N 141
Horn T 1405
Horne CHW 498
Horwich A 1623
Hospers GAP 184
Houlbrook S 219
House A 1261
Howe GR 1552
Howell SB 1047
Hoznek A 209
Hsieh C-c 1255 (L)
Hsu C-H 1510
Huang W-Q 1154
Huddart RA 863
Hughes SV 550
Hui A-M 50
Huikeshoven FJ 246
Hulscher TM 921
Humphreys JE 443
Hunai H 769
Hunter RD 620
Hussey HJ 56
Hutchins A-M 1659
Hyrynkangs K 614
Ianniello GP 707
Ichinose Y 928
Ide M 1607
Iizuka N 1026, 1209
Ikeda Y 887
Ikematsu S 701
Iles RK 1730
Illsley MC 650
Imbert T 1516
Imeson J 602, 1124
Inagaki N 56
Infante-Rivard C 1559
Inoue T 1488
Ishiguro S 769
Ishihara S 1394
Ishitsuka H 56
Ito K 1488
Ivaldi C 104
Iversen K 493
Izu R 847
Jackel S 1664
Jackman AL 792
Jackson SP 1702
Jacobs E 443
Jacobs I 279 (L)
Jacobs IJ 1287
James LA 467
Jantscheff P 1454
Janulis L 519
Jauch K-W 1216
Jaurand M-C 1147
Jia W 1468
Jick H 1556
Jin E 196
Jin R 319
Johansen C 89
Johansson M 826
Johnson JI 1401
Johnson PJ 1330
Johnsson A 1047
Johnston S 980
Joiner M 514
Jokinen K 614
Joly F 577
Jonasson JG 1715
Jones J 1344
Jones M 1084
Jordan VC 782
Jorna AS 1176
Jornvall H 171
Jouve M 1480
Joyeux I 1454
Juang B-T 1674
Julious SA 959
Jung R 874
Jungbluth AA 493
Kaanders JHAM 674
Kadomatsu K 701
Kadouri E 153
Author index 1773
British Journal of Cancer (2000) 83(12), 1771-1777
(c) 2000 Cancer Research Campaign
Kahlos K 880
Kaibuchi K 1168
Kakeji Y 986
Kambouchner M 1318
Kameyama M 769
Kanayama S 668
Kancherla R 360
Kane KF 550
Kannagi R 1681
Kantola S 614
Kanz L 458
Karesen R 1650
Karkavitsas N 1281
Karpus WJ 519
Kasinetz L 153
Kawaguchi H 609
Kawanami O 196
Kawano K 1096
Kawashima K 1394
Kaye JA 1556
Kaye SB 426
Kazumori H 1394
Keilholz U 6
Kelekis N 1281
Kelland LR 338
Kelleher DK 225
Kelsey A 602
Kennard DA 853
Kennedy MJ 1405
Kern FG 1154
Kerr KM 632
Keshoofy M 1249
Keshtgar M 1769 (BR)
Key TJ 95
Khan P 1599
Khuder SA 133 (L)
Kijima H 833
Kikuchi M 701
Kikuchi R 215
Kim CJ 1503
Kimura H 858
Kinnula V 880
Kinoshita J 887
Kinoshita Y 1394
Kirchner H 583
Kirk D 426
Kitamura K 609
Kitamura S 668
Kitano S 1096
Kitayama J 324
Kiviniemi M 1161
Kiyohara T 668
Kjartansson J 1715
Klamut HJ 998
Klastersky J 1128
Kleeberg UR 1351
Klein-Soyer C 346
Klijn JGM 384
Klimka A 252
Ko K 1039
Kobune-Fujiwara Y 769
Kocher T 204
Kogner P 171
Kohler JA 1124
Koike M 164
Koja K 928
Kolb D 493
Kolfschoten GM 921
Kollmannsberger C 458
Komatsu K 769
Kondo S 668
Konno R 1488
Kool M 366
Kornek G 454
Koskinen L-OD 826
Koukouraki S 1281
Koukourakis MI 219, 1281
Koumakis G 1128
Kreienberg R 1338
Kristjansdottir K 1715
Kroep JR 1069
Kroger N 874
Kroon BBR 1351
Kruczynski A 1516
Kruger W 874
Kuan A 91
Kubo N 215
Kuh D 964
Kulkarni S 91
Kuma K 1495
Kumai H 701
Kumar H 1425
Kundu SD 519
Kuninaka S 928
Kuper H 949, 1234
Kuriyama T 858
Kuroda S 1168
Kusiak I 1664
Kuwano H 609, 1033
Kvalheim G 1375
Kyrias G 1281
Kytola S 1020
La Vecchia C 689, 952, 1238
Laake K 1650
Labianca R 573
Lacornerie T 642
Ladner RD 792
Lafitte J-J 1128
Laiho P 1015
Lakari E 880
Lallemand E 1623
Lamb J 487, 800
Lambin P 514
Landman-Parker J 1617
Langman MJS 550
Lankelma J 375
Larsen RH 1375
Larsson B 307
Larsson C 1020
Launonen V 1015
Laurence V 1480
Laylor R 1202
le Maho Y 1055
Leal JE 969 (L)
Leblanc T 1617
Lee AS-G 750
Lee BL 1360
Lee C 519
Lee J-W 1674
Lee SM 1405
Lee-MacAry AE 1202
Lefebvre JL 1594
Lefrere-Belda M-A 209
Leibovitz I 463
Leigh DA 729
Lejars O 973
Lejeune FJ 1351
Lennon A 421
Leo F 569
Leonard P 418 (BR)
Leonard RCF 98, 135
Leslie MD 863
Levack P 98
Leverger G 973, 1617
Levi F 689, 952
Levin VA 588
Lewis C 438
Li G 1468
Li L-Y 1154
Li X 50, 407
Lie SO 1124
Lilge L 1110
Lim CK 935
Lin S 1762
Lin VCL 319
Lin X 1360
Lindgren A 391
Lindhofer H 261
Linet M 692
Ling Y-Z 74
Lingenfelser T 458
Lingnau K 1192
Lippman ME 1154
Lipworth L 1255 (L)
Lissia A 1707
Liu A 1154
Liu D 1473
Liu F 1256 (L)
Livne PM 463
Lloyd D 1118 (BR)
Lo Cunsolo C 1295
Locatelli MC 707
Lodge AJ 498
Logan RFA 127
Logue JP 620
Lohner R 874
Loncaster JA 620
Long BJ 74
Longnecker MP 404
Lonning PE 1650
Looijenga LHJ 729
Loos M 1192
Lopez-Michelena T 847
Lopez-Soriano FJ 526
Lopez-Soriano J 526
Los G 1047
Lotayef M 30
Lotze MT 506
Love SD 219
Loves WJP 1069
Luben R 121
Luboinski B 1594
Lucchi M 480
Lunec J 40
Luo C 1525
Lutz P 973
Lynch SF 1443
Lyng FM 1223
Lyng H 354
Ma I-F 1510
Macadam RCA 188
McAnulty RJ 650
McBride M 692
McClaren B 1612
McClay EF 16
McClay MET 16
McCrea PD 870
MacDonald N 279 (L)
McGee JO'D 750
McGown A 1599
McGuckin AG 40
Machin D 699 (L), 959
McIllmurray MB 447
McIntyre IG 992
McIntyre K 1405
McKendrick J 438
McKeown SR 1589
McKinney PA 127, 387
MacKinnon AC 941
McLeish R 1084
McLeskey SW 1154
McNutt M 112
McWilliam P 725
Maddau C 1462
Madsen EL 1256 (L)
Maehara Y 887, 986, 1168
Maelandsmo GM 743
Maeurer MJ 1192
Magdelenat H 1318
Maggi G 569
Magnani C 104
Magnenet P 642
Mah-Becherel MCM 346
Maisey NR 287
Maisonpierre PC 1154
Maitland NJ 1102
Maize JC 16
Makuuchi M 50
Malan A 1055
Malcolm AJ 40, 397
Malignant Melanoma
Cooperative Group 1351
Malik K 1583
Mancuso S 1762
Manfioletti G 1503
Mangili A 892
Manning J 473
Mannion EM 1432
Manusama ER 1176
Mao JH 566
Marandas P 1594
Marangoni E 514
Margison GP 69, 1702
Markham AF 188, 329
Marres HAM 674
Marsden B 602
Marti M-C 1637
Marty M 431, 577
Mason M 655, 1612
Massey A 146
Masters S 1268
Mathiot C 1454
Matsunaga T 1020
Matsuzawa Y 668
Matsuzuka F 1495
Matthay KK 1121
Matthews CS 270
Matthews E 519
Matthey B 252
Mauch C 1387
Maughan TS 447
Mead GM 1623
Mechelany-Corone C 715
Mechinaud F 973
Mehta J 91
Mehtali M 1454
Meijers-Heijboer EJ 384
Melamed R 1747
1774 Author index
British Journal of Cancer (2000) 83(12), 1771-1777 (c) 2000 Cancer Research Campaign
Melanoma Cooperative Group
1707
Mendes R 853
Menon U 279 (L)
Merlin J-L 1380
Mertins S 817
Metcalf JS 16
Metzger R 473
Miano R 1432
Miccoli P 1412
Michaeli D 443
Michaelis J 692
Michetti G 573
Michon J 973
Middleton H 602
Mignard D 431
Mikaelsdottir EK 1715
Millon R 1380
Millot F 973
Millward M 438
Milroy R 447
Min Y 1047
Minard V 973
Mineta H 775
Mirabelli D 104
Mirjolet J-F 1380
Misawa K 775
Misra V 998
Mitachi Y 141
Mitchell C 602
Mitchell H 1432
Mitchell P 91
Mittelman A 360
Mittler U 756
Miura K 775
Miura S 833
Miyamoto K 1209
Miyamoto S 1168
Miyauchi A 1495
Miyazaki M 887
Miyazaki Y 668
Miyoshi J 769
Mizukami T 1039
Mochizuki Y 1607
Mommen P 1128
Monks NR 267
Monroe L 16
Monson J 1425
Moonga BS 360
Moore J 63, 980
Morbidelli L 63
More L 397
Morel P 1637
Mori N 1026
Moriya T 858, 1488
Moriyama N 1394
Morris Jones P 602
Mothersill C 1223
Motoda H 1039
Mouret E 1318, 1480
MRC Testicular Tumour
Working Party 1623
Mucci LA 1234
Mueller J 1323
Mulatero C 1612
Mulder NH 184
Muller C 514
Munoz N 1238
Muramatsu H 701
Muramatsu T 701
Murata K 769
Murphy MFG 1231
Murray PV 1418
Muskiet F 594
Muslumanoglu M 737
Mussi A 480
Mutgi AB 133 (L)
Mysliwietz J 261
Nagano H 701
Nagao K 858
Nagasue N 1394
Nagawa H 324
Nakahara T 1607
Nakamura H 769
Nakamura M 833
Nakamura S 164, 1681
Napier MP 1274
Naraghi A 1274
Narayanan S 1532
Nass SJ 1688
Negri E 689
Nehme A 1360
Nejad-Sattari M 1367
Nekarda H 1323
Ness RB 404
Newell DR 267
Newton R 412
Ng S-C 83
Ng SW 498
Nicholl I 1643
Nicholson M 447
Nicolas A 1318
Nicolini A 1412
Ninane V 1128
Nischt R 1387
Nnane IP 74
Nocera M 715
Noci I 1722
Noferini D 1462
Noga SJ 1405
Noguchi T 215
Nogueira M 1139
Noma T 1026
Noppen C 204
Norman A 287
Normand C 561
Norton A 1418
Numico G 1437
Nyren O 391, 949
O'Brien MER 853, 1418
O'Brien PH 16
O'Byrne KJ 219
Oda M 701
Oda S 986
O'Doherty MJ 863
O'Dwyer ST 1344
Oevermann K 583
Ogino T 775
Ogmundsdottir HM 1715
Oh Y 756
Ohly KV 1405
Ohnishi K 1735
Ohnishi T 1735
Ohnishi Y 833
Ohno S 609, 1033
Ohtsu A 141
Ojima I 1762
Oka M 1026, 1209
Okamoto K 701, 833
Okamura K 1488
Okuyama T 1394
Olafsdottir K 1715
Old LJ 493
Olive PL 1525, 1740
Oliver RTD 1612, 1623
Olivotto M 1722
Ollivaud L 1243
Olsen JH 692
Olson E 1559
Olson J 588
Olson TA 1077
Ondrey FG 1367
Oosterhuis JW 729
Orr S 270
Orsetti B 1309
Osada T 324
Oshika Y 833
Osoba D 588
Osterreicher C 454
Osterwalder B 22
Ota I 1735
Ott RJ 287
Oudart H 1055
Owens C 602
Oya M 626
Ozbilen S 737
Ozcelik H 737
Paakko P 880
Padhani A 287
Paesmans M 1128
Paez de la Cadena M 1139
Page WF 1231
Paglierani M 1722
Palangie T 1480
Pallestrini E 1437
Palmer J 1003, 1009
Palmer RD 853
Palmieri G 1707
Pan T-L 1096
Papelard H 719
Paridaens R 594
Parikka M 614
Parker L 397
Parkins CS 811
Parle-McDermott A 725
Passos-Coehlo J-L 1405
Pastorino U 569, 935
Patael Y 153
Patterson AV 219
Patterson LH 1589
Patzelt T 583
Paul J 426
Paus E 743
Peacock JH 650
Peak S 63
Pearson ADJ 40
Pellegriti G 715
Penz M 454
Perel Y 973, 1617
Perrin D 1516
Perrone F 707
Perusinghe N 177
Peters AM 684
Peters GJ 1069
Petersen G 1643
Peto J 1565
Petridou E 1234
Pettersson F 239
Petti AR 1295
Peyrat J-P 1380
Peyroulet MC 973
Phillips P 588
Piazza E 707
Pierga J-Y 1480
Pignatelli F 1762
Pinedo HM 921, 1069
Pinkerton CR 338
Pirastu M 1707
Pirmoazen N 1249
Pirtskhalaishvili G 506
Pistolesi F 480
Plantaz D 973
Planting ASTh 22
Platt-Higgins A 1473
Pleau JM 1454
Poletti P 573
Ponce-de-Leon S 969 (L)
Poon TCW 1330
Popov Z 209
Porteous M 1643
Portnoy M 1110
Potten CS 1344
Potter DM 914
Pouillart P 1454, 1480
Povey AC 69
Powell JJ 1443
Powles R 91
Powles T 91
Prados MD 588
Praeuer HW 473
Price P 281, 1581 (BR)
Price T 146
Priest K 853
Pritchard J 602
Pritchard-Jones K 177
Pronk LC 22
Propper DJ 219
Puhakka A 880
Pujol J-L 8
Punnonen K 1161
Quadri A 573
Quietzsch D 458
Quinn LS 526
Quinn M 1556
Quinn MJ 278 (L)
Raderer M 454
Radvanyi F 209
Rafnar T 1715
Ragnarsson GB 1715
Rahman I 941
Rahman N 177
Rajamaki A 1161
Raleigh JA 674, 1525
Raleigh SM 935
Ramakrishnan S 1077
Ramirez J 104
Ramon Y 463
Rampling R 588
Randimbison L 952
Ranson M 1599
Rao S 146
Rapley EA 177
Raton JA 847
Rauth AM 998
Read A 278 (L)
Author index 1775
British Journal of Cancer (2000) 83(12), 1771-1777
(c) 2000 Cancer Research Campaign
Reed MWR 1061
Rees RC 1061
Reeve A 177
Reid M 397
Reigner B 22
Reis AC 1243
Renehan AG 1344
Reynolds CP 1121
Rialland X 973
Ricci I 1437
Richardson G 438
Rijken PFJW 674
Rindi G 952
Rindsfuser M 637
Rintoul R 941
Rischin D 438
Ristori E 1503
Rittling SR 156
Riva A 532, 1762
Rizvi I 1544
Robbiati SF 707
Roberts DD 298
Roberts I 1309
Roberts JT 1623
Robinson PA 188
Rochlitz C 1454
Rocmans P 569
Roder RJ 35
Rodriguez-Berrocal FJ 1139
Roelofs H 729
Rofstad EK 354
Rohner S 1637
Roman E 692
Romerio F 532
Ronco G 1462
Roovers RC 252
Rosati B 1722
Rosenberg R 35, 1323
Rosenfeld RG 756
Rosenne E 1630
Rosenthal AN 1287
Roshan MK 1249
Ross EL 1202
Ross JA 700 (L), 1443
Ross PJ 146
Rosso R 1437
Roth AD 1637
Roth JA 473
Roth S 1015
Rothman VL 298
Rotmensz N 1243
Rougier P 431
Rowlinson-Busza G 232, 684
Royer-Pokora B 177
Rubie H 973
Rudland P 1084
Rudland PS 1473
Ruiter DJ 1351
Rumi MAK 1394
Runnebaum IB 1338
Rustin GJS 1274
Ryan A 1287
Ryan C 853
Rylander E 307
S-1 Cooperative Colorectal
Carcinoma Study Group 141
Sackett D 817
Saeki H 609
Sage RD 134 (L)
Sahin AA 870
Saidi F 1249
Saily M 880
Saini A 853
Saip P 737
Saito S 324
Saito T 1168
Sakata Y 141
Sakuma S 701
Sala E 121
Salehian P 1249
Salles B 514
Sallstrom J 307
Salminen E 1161
Salo T 614
Salom AV 1480
Salovaara R 1015
Salup RR 506
Samaritani E 480
Sancho-Garnier H 1594
Sandler DP 404
Sandor V 817
Sanguineti G 1437
Santelli A 1437
Sarela AI 188
Sasano H 1488
Sasieni P 561
Saso R 91
Sastre-Garau X 1318, 1454
Sathyamoorthy N 333
Sato H 1394
Sato S 1488
Sauroja I 1161
Sausville E 817
Sausville EA 1401
Savage P 980
Sawa N 833
Scambia G 1762
Schaison G 1617
Schally AV 906
Schey S 1268
Schlumberger M 715
Schmerber J 1128
Schmitt B 261
Schmitt E 1192
Schneider PM 473
Scholfield DP 443
Scholl S 1480
Schrader A 583
Schraffordt Koops H 184, 1351
Schroeder M 458
Schultz-Thater E 204
Schultze-Seemann W 637
Schulz WA 626
Schumacher V 177
Schurz H 782
Scott D 1136
Scott KA 1538
Scott L 800
Scott PAE 63
Screpanti I 1503
Sculier J-P 1128
Scurry JP 1659
Seabra L 1084
Seeghers M 594
Segnan N 1462
Selker RG 588
Senderowicz A 817
Seo Y-C 750
Seow-Choen F 750
Sepehr A 1249
Sethi T 941
Severi G 1243
Severson RK 700 (L)
Seymour CB 1223
Seynaeve C 384
Seynhaeve ALB 1176
Shakhar G 1630, 1747
Shakhar K 1630
Shalet SM 1344
Shamash J 1258 (L)
Shannon R 602
Shapiro W 588
Sharp L 127
Sharpe CR 112
Sharrard RM 1102
Sheer D 40
Sheffield E 69
Shelling A 177
Shen GH 196
Shepherd J 1003, 1009
Shi Y-Z 50
Shibata Y 324
Shimizu H 196
Shinohara H 1039
Shinomura Y 668
Shohtsu A 1607
Shouman T 30
Shtriker A 463
Shurin GV 506
Shurin MR 506
Sibson DR 1473
Sidi AA 463
Siebert R 743
Siersema PD 539
Signorello LB 391
Sikic BI 892
Silvester J 935
Siman SE 69
Simms MS 443
Simpson A 426
Singh R 1654
Skeen J 177
Skinner J 692
Skovlund E 1650
Skytthe A 1231
Smeland EB 743
Smid-Koopman E 246
Smith A 487
Smith AJ 366
Smith DR 761
Smith GL 1432
Smith IE 853, 1268, 1418
Smith J 438
Smith S 800
Snary D 1202
Soderstrom, K-O 1161
Soini Y 614, 880
Sommelet D 1617
Souberbielle BE 853
Souhami R 1771 (BR)
Soussi T 1418
South and West Regional Cancer
Organisation Tumour Panel for
Head and Neck Cancer 421
Spagnoli GC 204
Sparidans R 375
Sparreboom A 22
Spence A 588
Spinelli C 1412
Spreadborough A 1136
Spreckley K 992
Squiban P 1454
Staniforth JN 935
Stark DPH 1261
Steele F 935
Steinbauer M 1216
Stenning SP 1623
Stephan JL 973
Stephen R 1183
Stephens RJ 447
Stevens MFG 270
Stevenson AJ 329
Steward M 498
Stewart AFR 134 (L)
Stewart JSW 232, 684
Stewart PM 550
Stewart THM 134 (L)
Stockert E 493
Stoeltzing O 473
Stoppa-Lyonnet D 1318
Straatman H 1351
Strand A 307
Stratton MR 177
Strazzullo M 1707
Stromboni-Luboinski M 1594
Suchy B 1664
Suciu S 6, 1351
Suetsugu H 1394
Sugimachi K 141, 609, 887,
986, 1033
Sugisaki Y 196
Summerhayes M 1268
Sun B 906
Sunami E 324
Sundfor K 354
Suzuki I 775
Suzuki N 769
Suzuki T 1488
Swansbury GJ 91
Swerdlow AJ 964
Symonds P 566
Syrigos KN 232, 684
Szepeshazi K 906
Tabeta H 858
Tabrizi SN 1659
Taddei GL 1722
Taguchi T 141
Tahir SK 83
Takahashi A 1735
Takahashi I 986
Takahashi R 1039
Takahashi W 1607
Takano T 1495
Takayama T 50
Takebayashi S 775
Takeno S 215
Takiguchi Y 858
Talamini R 689, 1238
Talbot DC 219
Tamaoki T 1039
Tan PH 319
Tanaka Y 56
Tanda F 1707
Tang W-Y 761
Tangoku A 1026, 1209
Tartour E 1454
Tatematsu M 1681
Tatsuta M 769
1776 Author index
British Journal of Cancer (2000) 83(12), 1771-1777 (c) 2000 Cancer Research Campaign
Tavani A 1238
Tavassoli M 1418
Taylor C 1565
Taylor CPF 40
Taylor M 219
Taylor R 602
Taylor-Papadimitriou J 1202
Te V-C 952
Teh B 1003, 1009
ten Hagen TLM 1176
Terlouw EM 539
Terracini B 104
Thatcher N 447
Theillet C 1309
Theodorsson E 171
Thews O 225
Thiery JP 209
Thiriaux J 1128
Thomassen J 1405
Thorban S 35
Thyss A 973
Tiebosch ATMG 184
Tighe O 725
Tilanus HW 539
Timmer-Bosscha H 184
Timothy AR 863
Tischler T 463
Tisdale MJ 56
Tobias JS 971 (BR)
Todorov PT 56
Toh Y 928, 1033
Tohari S 750
Toi M 164
Tokunaga T 833
Tolis C 1069
Tollenaar RAEM 719
Tominaga T 164
Tomozawa S 324
Toner G 438
Tonetti DA 782
Tonini GP 1295
Toniolo PG 1255 (L)
Topping K 1425
Tozer GM 662, 811
Treleaven J 91
Trichopoulos D 949, 1234
Trillet-Lenoir V 431
Trock BJ 1688
Trope CG 354
Truchetet F 1243
Tsuchida T 833
Tsuno NH 324
Tsuruo T 324
Tuckwell N 853
Turkmen S 737
Turley H 63
Turuguet D 104
Tuszynski GP 298
Twelves C 22
Tynes T 692
Uchida Y 215
Uciechowski P 1664
Ueda Y 775
Ueno T 164
Ueyama Y 833
Uges DRA 594
United Kingdom Childhood
Cancer Study Investigators
1573
United Kingdom Children's
Cancer Study Group 40, 602
United Kingdom Co-ordinating
Committee 294
United Kingdom NSCLC
Gemcitabine Group 447
Underwood M 426
Uri N 1696
Usel M 104
Uster PS 232, 684
Vaccarella S 1238
Vaganay-Juery S 514
Vaidya JS 1769 (BR)
Vaittinen P 407
Valencak J 454
van Ballegooijen M 561
Van Buuren I 594
van Deemter L 366
van Dekken H 539
van den Akker-van Marie E 561
van den Hoogen FJA 674
van der Graaf WTA 594
van der Kogel AJ 674
Van der Pavert I 899
Van der Schueren B 899
van der Wall E 1405
van Eijk R 719
van Etten B 1176
Van Geel AN 569
van Geloof WL 1351
van Groeningen CJ 1069
van Hillegersberg R 539
van Muijen GNP 1351
van Oosterom AT 594
van Tiel ST 1176
Van Waes C 1367
Vantard M 544
Varcoe S 219
Varley JM 467
Vasey P 22
Vaughan M 980
Vaupel P 225
Vazina A 463
Veitia R 544
Verhoog LC 384
Verkasalo PK 692, 1231
Vernillet L 146
Verschoyle RD 935
Verweij J 22
Vick NA 588
Vidarsson H 1715
Vincent F 346
Vincent-Salomon A 1318
Vitale V 1437
Vliet Vlieland TPM 719
Vogelsang G 1405
Vogl FD 1338
von Eyben FE 1256 (L)
Voorn DA 1069
Vorbeck F 454
Vorwerk P 756
Vu P 1650
Vujanic GM 602
Wadsworth M 964
Walker M 1646
Walker SJ 959
Walter W 1192
Walters M 1003, 1009
Walz A 261
Wang T-M 1510
Wang TTY 333
Wanke E 1722
Ward TH 69
Warenius HM 1084
Warn RM 1147
Warren R 121
Watanabe M 609, 1033
Watanabe R 858
Waters C 941
Webb A 287, 853, 980
Webley SD 792
Wegerer S 473
Weiderpass E 949
Weirich A 177
Welch A 121
Weltin D 642
Wennerberg J 775
West CML 620, 1702
Westhoff C 1552
Wetterauer U 637
Wex H 756
Wheatley DN 800
White DJ 1659
Whitehouse A 329
Whiteman DC 1231
Wibault P 1594
Wielinga PR 375
Wilander E 307
Wild R 1077
Wilkinson PM 1623
Wilkinson RW 1202
Williamson B 493
Wilson BC 1110
Wilson CR 1702
Wilson GD 30
Wilson JHP 539
Wollenberg B 261
Wu Y 156
Wu-Wong JR 360
Wijffels KIEM 674
Yaacobi Y 463
Yajima A 1488
Yamamoto K 1026
Yamamura Y 1681
Yamaoka Y 1039
Yamazaki H 833
Yang C-S 1674
Yang T 519
Yang YM 360
Yano A 701
Yap W-M 761
Yarnold JR 650
Yasuda M 1033
Yasuda S 1607
Yazdanbod M 1249
Yazici H 737
Yazumi S 1039
Ye W 949
Yeh K-H 1510
Yeh S-H 1510
Yokoyama H 1096
Yoshida H 1209, 1495
Yoshida T 1039
Yoshikawa K 1039
Yoshimura K 1209
Yoshino S 1026, 1209
Yu J 1154
Yue N 588
Yule R 1565
Yung WKA 588
Zacharias C 1128
Zafrani B 1318
Zajac P 204
Zaknoen S 588
Zander AR 874
Zani M 1503
Zeelenberg I 1047
Zehavi M 463
Zeidler R 261
Zella D 532
Zhang H-T 63
Zhang Q 1360
Zhou J 1039
Zhu L 1255 (L)
Ziche M 63
Ziegler I 261
Zigrino P 1387
Author index 1777
British Journal of Cancer (2000) 83(12), 1771-1777
(c) 2000 Cancer Research Campaign

				

